# The complete plastid genome sequence of *Peucedanum hakuunense* Nakai (Apiaceae), an endemic and rare species in Korea

**DOI:** 10.1080/23802359.2022.2069522

**Published:** 2022-05-05

**Authors:** Ho Jun Joh, Hyun-Seung Park, Jong-Soo Kang, Jee Young Park, Tae-Jin Yang

**Affiliations:** Department of Agriculture, Forestry and Bioresources, Plant Genomics and Breeding Institute, College of Agriculture and Life Sciences, Seoul National University, Seoul, Republic of Korea

**Keywords:** *Peucedanum hakuunense*, plastid genome (plastome), phylogenetic analysis, Apiaceae

## Abstract

*Peucedanum hakuunense* Nakai is one of the rare species in the Korean Peninsula. This study characterized the complete plastid genome (plastome) sequence of *P. hakuunense* by *de novo* assembly with next-generation sequencing data. The complete plastome of *P. hakuunense* is 147,426 bp in length with a typical quadripartite structure comprising a large single-copy region of 91,915 bp, a small single-copy region of 17,425 bp, and two inverted repeat regions of 19,043 bp in length. The plastome of *P. hakuunense* is composed of 85 protein-coding genes, 36 tRNA genes, and 8 rRNA genes. The phylogenetic analysis revealed that two *Peucedanum* species formed an independent subclade, sister to the subclade of *Angelica* species within the tribe Selineae.

*Peucedanum hakuunense* Nakai (common name: Baekun hog fennel) is a member of the genus *Peucedanum* distributed only in the Korean Peninsula. This species was first reported by Nakai (1939) (Lee 1982) has a morphological characteristic of 30 ∼ 40 cm in plant height, with a narrow pinnate tri-ternate radical leaf of 10–18 cm in length with a wide triangle lobe bipinnate leaf. It has a compound umbel with 15–30 individual white flower stalks with purple stamen. Mericarp fruit is of flat oval shape with 4.5 mm length and 2.5 mm width with three veins on the back with white wings on the edge. Of all, the *Peucedanum* species discovered within the Korean Peninsula, *P. hakuunense* is one of the rare species (Na et al. 2017). Although it was considered an endemic and rare species in Korea, *P. hakuunense* was poorly studied in genetic and genomic approaches. Here we report the complete plastid genome sequence of *P. hakuunense* to facilitate taxonomic and phylogenetic approaches from phylogenetic inference on the genus *Peucedanum* and advance the understanding of the phylogenetic relationship in the family Apiaceae.

*Peucedanum hakuunense* specimen was collected from Baekun Mountain located in Kwangyang City, Jeollanam-do Province (35°1′42″ N, 127°36′13″ E, Nambu University Forest, Seoul National University). DNA was extracted using the CTAB protocol (Allen et al. 2006). The specimen's isolated genomic DNA was deposited at the National Institute of Biological Resources (42, Hwangyeong-ro, Seo-gu, Incheon 22689, Korea; Contact person: Yoon-Jeong Park, byj6019@korea.kr) under the specimen number of NIBRGR0000635909. About 1 μg of purified DNA was sequenced using the Illumina Miseq platform (Illumina Inc, San Diego, CA, United States). Approximately 1.2 Gbps paired-end sequencing data were obtained for *P. hakuunense* sample. High-quality reads were used for *de novo* assembly on the plastome with low coverage whole genome sequencing (dnaLCW) method (Kim et al. 2015). In brief, the raw reads were trimmed using a trimming tool and then assembled into contigs using the CLC assembly tool (ver. 4.06 beta, CLC Inc, Aarhus, Denmark). Using a previously reported plastome sequence of *Peucedanum japonicum* (NC_034644) as a reference, the assembled contigs with high similarity to the reference sequence were then extracted using MUMmer (Kurtz et al. 2004) and eventually assembled into a whole plastome sequence. Gene annotation on the draft plastome sequence of *P. hakuunense* was performed using GeSeq (Tillich et al. 2017) and Artemis (Rutherford et al. 2000) and then manually curated.

The plastome of *P. hakuunense* was 147,426 bp in length with 37.52% of GC content. The plastome structure was typical quadripartite featuring two copies of inverted regions (IRa and IRb) with 19,043 bp each, a large single-copy region with 91,915 bp, and a small single-copy region with 17,425 bp in length. A total of 129 genes, including 85 protein-coding genes, 36 tRNA genes, and 8 rRNA genes, were found in the plastome of *P. hakuunense*. The phylogenetic analysis was performed with representative species in the family Apiaceae. The plastome sequences of 12 Apiaceae species were downloaded from NCBI GenBank (https://www.ncbi.nlm.nih.gov/genbank/), as well as a complete plastome sequence of *Panax ginseng* in Araliaceae was included as an outgroup. A total of 78 plastid genes, shared by all 14 species, were used for phylogenetic reconstruction (Figure 1). Each CDS region of plastid genes was extracted by Feature Extract (Wernersson 2005) and then concatenated into a single matrix. The maximum-likelihood (Chumley et al. 2006) tree was reconstructed using raxmlGUI 2.0 (Edler et al. 2021) with 1000 bootstrap replicates under the GTR + G+I model selected by jModeltest v. 2.1.5 (Darriba et al. 2012). As a result, the ML tree has demonstrated that *P. hakuunenese* was sister to *Peucedanum terebinthaceum*, followed by the subclade of *Angelica* species (*A. gigas and A. decursiva)* forming a clade under the tribe Selineae. The phylogenetic relationships within the subfamily Apioideae were consistent with the phylogenetic relationships in a previous study (Wen et al. 2021). The newly reported plastid genome sequence of *P. hakuunense* will be helpful to advance our current understanding of phylogenetic relationships between the genus *Peucedanum* and the family Apiaceae.

**Figure 1.**
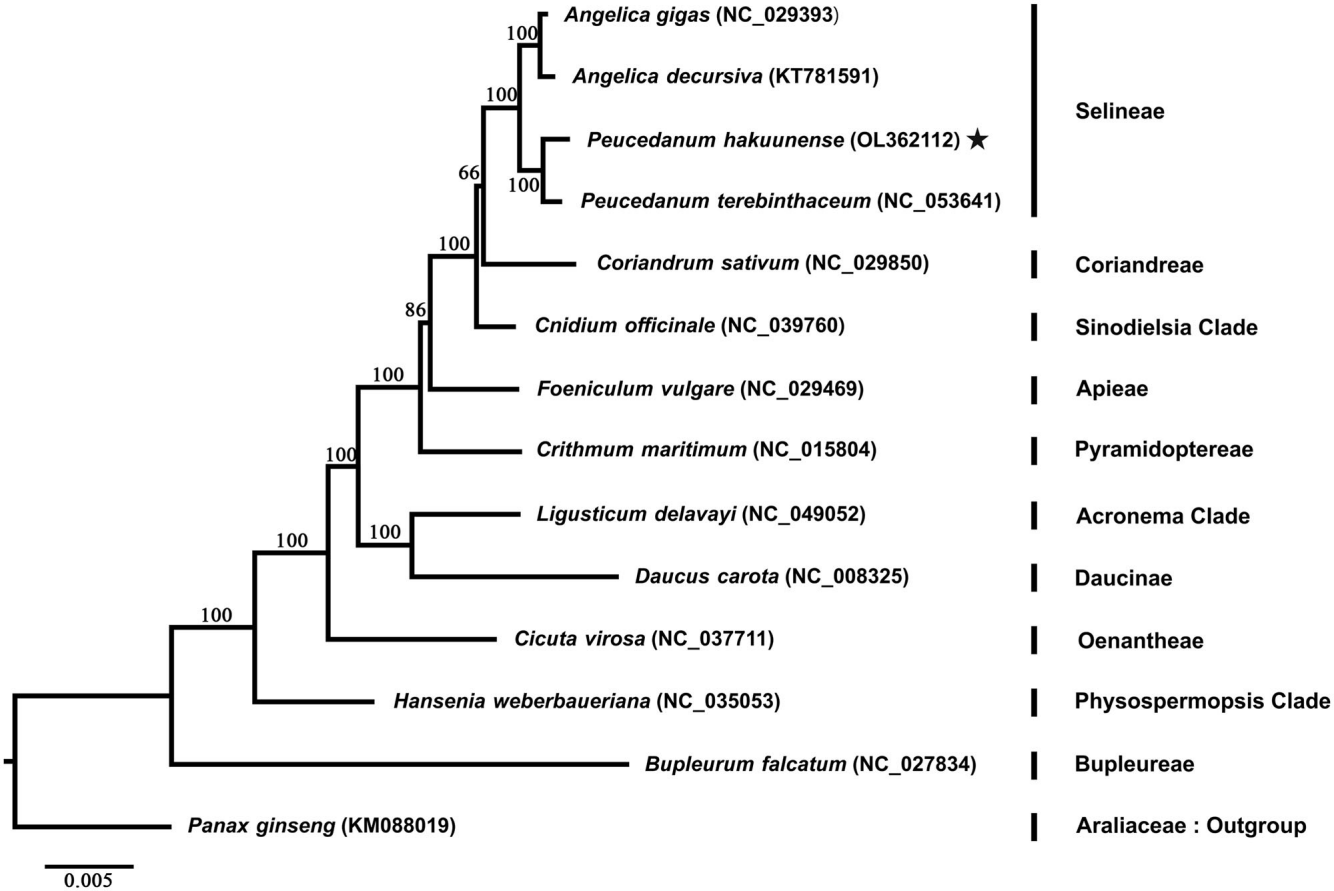
Phylogenetic analysis of Apiaceae based on 78 plastid genes from 14 species. Star indicates the newly generated plastome of *P. hakuuense* in this study. A phylogenetic tree was reconstructed using the maximum-likelihood method in raxmlGUI 2.0 with 1000 bootstrap replicates. Numbers at the node are the bootstrap supporting value calculated from the ML method. The plastome sequence of *Panax ginseng* from Araliaceae was included as an outgroup.

## Data Availability

The plastid genome sequence data supporting this study’s finding is available in NCBI GenBank under the accession number OL362112. The associated BioProject, SRA, and Bio-sample numbers are PRJNA775900, SRR16611559, and SAMN22635847, respectively.
